# Equity, justice and the SDGs: lessons learnt from two decades of INEA scholarship

**DOI:** 10.1007/s10784-022-09563-w

**Published:** 2022-04-05

**Authors:** Joyeeta Gupta, Aarti Gupta, Courtney Vegelin

**Affiliations:** 1 Inclusive Development Programme Group, Amsterdam Institute for Social Science Research of the University of Amsterdam, Amsterdam, The Netherlands; 2 IHE Delft Institute for Water Management, Delft, The Netherlands; 3 Environmental Policy Group of the Social Sciences Department, Wageningen University, Wageningen, The Netherlands; 4 Earth System Governance Project, Utrecht, The Netherlands; 5 Insert Governance and Inclusive Development Programme Group, The Amsterdam Institute for Social Science Research of the University of Amsterdam, Amsterdam, The Netherlands

**Keywords:** Sustainable Development Goals (SDGs), Equity, Justice, Access, Allocation, International Environmental Agreements

## Abstract

Environmental justice issues have been incrementally but consistently covered within this journal in the last two decades. This article reviews theoretical and empirical approaches to justice in INEA scholarship in order to identify trends and draw lessons for the interpretation and implementation of the 2030 Agenda and for living within environmental limits. Our review traces how justice considerations were initially covered within new institutionalist scholarship on collective action and social practices, to conceptualizing justice as ‘access and allocation’, to newer notions of planetary justice. We link these trends to scholarship on diverse epistemologies and typologies of justice, including conservative, corrective, distributive and procedural justice, and examine their operationalization within the empirical domains of climate, water and sustainable development. In concluding, we draw out implications for the 2030 Sustainable Development Agenda. We argue that a just approach is essential to living within environmental limits, with greater synergies needed between collective action and social practice approaches. While justice can be unpacked for practical and political reasons into access and allocation, we find that (procedural) access considerations are more politically palatable in practice than a concern with allocation (distributive justice), which remains much more contested. As such, dominant approaches promote ‘conservative’ or thin market-based notions of justice. We conclude by noting that just allocation is a precondition to just access. A failure to prioritize and achieve more corrective and distributive forms of justice will, without doubt, contribute to exacerbating global ecological degradation.

## Introduction

As part of the Special Issue celebrating 20 years of scholarship within *International Environmental Agreements: Politics, Law and Economics (INEA),* this paper focuses on equity, justice and the Sustainable Development Goals (SDGs). The history of justice including equity scholarship within (INEA) runs parallel to its history within the International Human Dimensions Programme’s (IHDP) project on Institutional Dimensions of Global Environmental Change (IDGEC) (1997–2007) and its follow-up project, Earth System Governance (ESG) (2008–present).

In the 1990s, beyond relevant legal and development studies scholarship, justice scholarship was looked down on as ‘normative’. When IHDP-IDGEC scholars established INEA, they hoped to encourage interdisciplinary scholarship on international environmental agreements. At that time, scholarship on environmental law and environmental agreements was just beginning and scholars were afraid that the pace of globalization would far exceed the ability of states to negotiate these agreements in an effective and fair manner (see Sand and McGee, this issue). IDGEC focused on how institutions addressed global change problems, how effective they were and ‘the extent to which outcomes conform to normative standards relating to matters of distribution’ (Young et al., 1999, p. 19); this was covered in INEA’s first issue in 2001.

With the adoption of the Millennium Development Goals (MDGs) (UN Secretary-General, 2001), certain justice issues (e.g. access) were prioritized at the global level. When the IDGEC project ended in 2008, its follow-up project, Earth System Governance (ESG), included justice as ‘access and allocation’. This conceptualization of justice could be scientifically observed, measured and analysed, engage ESG scholars, and be politically palatable. INEA published the ESG Science Plan in 2010 and reviewed access and allocation research in 2020. In 2015, with the adoption of the Sustainable Development Goals (SDGs) (UNGA, 2015), the time appeared ripe for an explicit focus on justice. The ESG’s new Science Plan (ESG, 2018) adopted inequality, justice and equity as central conceptual lenses to ESG scholarship and practice.

The scholarship on justice is scattered through many disciplinary journals on ethics, philosophy, law, politics, sociology and anthropology. While there is growing scholarship on global (e.g. Brock, 2021), environmental and planetary justice (e.g. Biermann & Kalfagianni, 2020), there are no scoping reviews of this scholarship with respect to international environmental agreements. This is the gap that our article fills. We review how justice has been analysed conceptually and empirically within the pages of this journal, given INEA’s unique global niche and focus on international environmental agreements.

Against this background, we ask: What are the trends in the development of theoretical and empirical approaches to justice and what lessons can be drawn for the interpretation and implementation of the 2030 Agenda and for living within environmental limits?

We examine 20 years of INEA scholarship on environmental justice. We selected 150 papers that referred directly or indirectly to equity, distributive issues, access and allocation, or justice. We clustered this literature into three storylines. We examined the justice issues in papers published in other journals to see how they fitted or contrasted with these storylines. We also assessed the references to equity and justice in the other papers reviewing INEA scholarship in this Special Issue. Below we discuss justice conceptualizations (Sect. 2), key themes and empirical results (Sects. 3 and 4), analyse the material (Sect. 5) and draw conclusions.

Important to note is that INEA focused on *environmental* justice issues because of the journal’s scope. Since 2015, INEA papers also examine social–ecological justice resulting from the 2030 Agenda’s explicit requirement to link social, ecological and economic issues. INEA papers are generally more empirical than philosophical and theoretical, and cover transboundary to global justice rather than local social justice covered in the broader justice literature.

## Conceptualizing justice: theoretical approaches

Four INEA papers have advanced conceptual thinking on justice. First, Young (2001) examined institutions and implicitly their implications for distributive justice. The second unpacked justice into access and allocation (Biermann et al., 2010; Gupta & Lebel, 2010). The third and fourth linked access and allocation to diverse epistemological approaches to realizing justice (Gupta & Lebel, 2020; Kalfagianni & Meisch, 2020).

### Conceptualizing justice: collective action and social practice models

INEA’s first issue set a pragmatic tone for discussing justice. Within the New Institutionalism School, Young (2001) differentiated between *collective action* and *social practice models* to explain and assess two behavioural pathways which influence negotiations to address the tragedy of the commons. He did not intend to nor present a justice framework. Nonetheless, we include it here because it showed how and why different models had different distributive outcomes with the social practice models having a greater chance of fair outcomes. Collective action models follow the ‘logic of consequences’. They are utilitarian and rational, weigh costs and benefits, prioritize agents over structure, see rules as constraints, maximize the net benefits a regime gives to the parties and take a political engineering approach. Relevant scholarship covers a narrow set of interests and constraints using a small family of ‘compact and parsimonious’ approaches (Young, 2001: 15) (including game theory, prisoner’s dilemma, cost–benefit analysis). These models lead to some form of ‘market justice’.

In contrast, social-practice models focus on the ‘logic of appropriateness’ (March & Olsen, 1998), on legitimacy, authority and norms (Young, 2001: 13). Here, actors display fair and appropriate behavioural patterns through socialization and routinization. Structure trumps agents, actors internalize rules, and such internalization shapes their identity. Scholarship on this is vast and diverse, and context is central. These models include more egalitarian forms of justice arising from the choice of norms (see Table 1). Young concludes that although synthesizing the two approaches is desirable, in the short-term one should understand why and when one approach prevails over the other.

### Conceptualizing justice: access and allocation in earth system governance

The next turn in justice scholarship was in 2010 when Gupta and Lebel (2010) went beyond neutrally examining behavioural pathways to unpacking justice in multidisciplinary terms of Access and Allocation within the ESG project. First, they explained how environmental resources can be distinguished between those required to meet basic needs/rights (access) versus those pertaining to distributing the surplus resources, risks and responsibilities (allocation). Both processes are organized through resource governance institutions which distribute: benefits and opportunities to users; responsibilities to managers; and risks and burdens to the affected. Such allocation is affected by social relations, processes and categories; capabilities, incentives and entitlements; and decisions are influenced by interests, power and beliefs. Second, they identified how different disciplines frame questions in relation to access and allocation in local to global environmental governance and how together they enable an interdisciplinary perspective. Ten years later, a Special Issue reviewed the literature on these concepts (see Sect. 3.1).

### Conceptualizing justice: epistemological approaches and ethical lenses

A third contribution (Kalfagianni & Meisch, 2020) focused on epistemological approaches to justice within the study and practice of environmental institutions. It identified three ethical lenses: conservative (i.e. ‘what can a person legitimately expect?’) versus ideal (i.e. ‘what should a person get?’); corrective (i.e. ‘has there been harm done and how can it be corrected?’) and distributive (i.e. ‘how can distributive issues be addressed?’); and procedural (i.e. ‘which processes can or are being used to distribute benefits?’) versus substantive (i.e. ‘what should a just social order look like?’).

Through these lenses, they examined scholarship (including 62 INEA papers) on access and allocation. They concluded that scholarly papers on access focus on ideal, rather than conservative justice; many synergistically examine corrective-distributive issues; and there is a greater focus on procedural rather than substantive justice (see also Sand and McGee this issue). Paradoxically, the literature on allocation focuses more on conservative justice—taking the rules as given and then exploring their impacts, including costs and benefits—and is less focused on corrective (see Sect. 3.2) than on distributive justice.

While Young observed the behavioural pathways that led to two different models of institutions with different distributional impacts, Gupta and Lebel (2010) implicitly argued that both models need to be assessed in terms of issues of access and allocation of resources, risks and responsibilities, while Kalfagianni and Meisch went deeper into the ethical roots of justice and used justice lenses to assess the empirical scholarship within INEA on access and allocation (see Sect. 5.3).

## Operationalizing justice: access and allocation, no harm and the green economy

### Access and allocation in practice: lessons from a decade of scholarship

The above conceptualizations have influenced or been influenced by INEA papers on justice. A Special Issue (2020) reviewed the literature on access and allocation issues and their synonyms (e.g. equity, justice). It examined the global economic system, trade, investment, climate change, food, water and waste. Three messages emerged.

First, ‘access’ and ‘allocation’ provide a practical starting point for unpacking and analysing justice concerns. ‘Access’ aligns with the social goals in the MDGs/SDGs, human rights and the social floors in the doughnut approach (Raworth, 2012). Allocation issues are more contested but are linked to the SDG on inequality (SDG 10), the common but differentiated responsibilities principle and the no harm/liability principle in international law. Thus both scholarship and politics are converging at least rhetorically on recognizing the need for a minimum threshold for humans (i.e. access) in an ‘ideal’ justice framing. However, allocation—both corrective (see Sect. 3.2) and distributive is contested, and framed ‘conservatively’ leaning towards collective action and market mechanisms (e.g. for allocating carbon credits [see Pouw et al., this Special Issue] and access to water, food and energy are subject to cost-recovery principles).

Second, many authors prefer to use equity and justice rather than access or allocation. Different disciplines prefer different concepts. Moreover, some issues cannot be neatly addressed in access and allocation terms; for example, it does not make sense to literally discuss access to biodiversity and allocation of biodiversity.

**Third, issues of access cannot be addressed without solving issues of allocation**. This implies that the current global focus on meeting minimum needs under the SDGs paternalistically through pro-poor policies will fail if allocation models are not also redesigned; this has been the major contribution of the access and allocation approach (see Sect. 5.5 for details).

### Corrective justice in practice: the ‘No Significant Harm’ principle

The de facto allocation of risks and harm (corrective justice) through current production and consumption processes is understudied. Moreover, some argue that harm and vulnerability to harm is politically created (Grecksch & Klöck, 2020). These require compensation and redress. Nationally, harm is dealt with under tort (non-contractual), administrative and criminal law. Internationally, the ‘no harm principle’ from the 1938 Trail Smelter arbitration between the USA and Canada has been further developed in water law.

INEA’s No Significant Harm Special Issue of 2020 shows that harm must be seen as: (a) ‘significant’, and (b) reflect lack of due diligence by the party causing the harm (Moynihan & Magsig, 2020). Yet this is difficult to (dis)prove in practice (McIntyre, 2020). Furthermore, (c) while the ‘no harm principle’ may contradict the equity principle (as, for instance, the party who has to reduce its water consumption to make water available to the other party is thus harmed by this action), the two may be mutually compatible (Tanzi, 2020). In addition, (d) the no harm principle supports human rights principles (Spijkers, 2020), and (e) it replaces the principle of absolute territorial sovereignty with that of limited territorial sovereignty. Further, (f) it requires states to conduct Environmental Impact Assessments of large projects, ensure minimum flows in rivers (Tignino & Bréthaut, 2020), and notify and consult with other states (Schmeier, 2020). Finally, (g) in respect to the no harm principle, courts are increasingly holding states responsible for the acts of domestic private actors (Rieu-Clarke, 2020).

Despite these trends seen in transboundary water law and scholarship, global progress on the no harm principle has been limited. This may be because such harm is complex and can be multi-directional. The source and impact of harm is diffuse and involves multiple actors and levels of governance. Moreover, harm is not just immediate but creeping and cumulative with a spatial/temporal dimension involving faraway places, the past and the future. Drawing the line between insignificant and significant harm is challenging. As harm is cumulative, the tolerance for harm decreases over time as we reach local to planetary boundaries (Gupta & Schmeier, 2020). In addition, the no harm principle, liability and compensation are not covered in the global climate and biodiversity treaties or in the 2030 Agenda. It is lost in translation in the principles of ‘common but differentiated responsibilities and respective capabilities’, ‘loss and damage’ in the climate change regime and the ‘shared responsibility’ concept in the 2030 Agenda. Negotiations on the Global Pact on the Environment which included harm and liability initiated by France have stalled. While the International Law Commission was working on this issue, they appear reluctant stating (UNGA, 2013: para. 168):

“(a)Work on this topic will proceed in a manner so as not to interfere with relevant political negotiations, including those on climate change, ozone depletion, and long-range transboundary air pollution. The topic will not deal with, but is also without prejudice to, questions such as the liability of States and their nationals, the polluter-pays-principle, the precautionary principle, common but differentiated responsibilities, and the transfer of funds and technology to developing countries, including intellectual property rights.”

The politics around this principle allows countries to pollute with impunity. However, the menu of legal and policy principles and instruments for such justice is vast. Court cases worldwide on water, climate and environmental justice issues are focusing on corrective justice calling, e.g. on states (e.g. The Netherlands) and oil multinationals (e.g. Shell) to reduce their emissions. Simultaneously, multinationals are suing for compensation from governments for closing down their fossil fuel companies. It is unclear what the net effect of these cases will be on setting justice precedents and on contributing to the further conceptualization of justice.

### International environmental justice and a green global economy

Resources are often allocated through the market or market mechanisms. Given that a driver of environmental degradation is the economic system (UNEP, 2019), justice requires us to examine development discourses: sustainable development, green economy and inclusive development. The INEA Special Issue on the Green Global Economy assesses whether the ‘green economy’ implies a shallow or deep engagement with justice. It identifies three approaches: (a) a thin green economy with a market justice approach; (b) a moderate green economy with egalitarian justice; and (c) thick green economy with structural justice (Okereke & Ehresman, 2015).

Many examples show that market justice prevails: A case study on a dam in Brazil shows that the green economy is used to justify technocratic governance serving capitalism and ignoring social justice (Bratman, 2015). A US study finds that implementing a green economy in marginalized urban areas created tensions between the people and policymakers, resolvable only through inclusive participation (McKendry & Janos, 2015). The message is that a green economy combined with a market approach fits in the collective action model, enriching investors while marginalizing social issues. This confirms the finding that without just allocation mechanisms, access will also fail.

## Empirical domains: climate, water, the 2030 Agenda

### Climate change: moving to a post-equity phase?

Recent INEA scholarship analyses whether and how substantive justice is being operationalized in climate policy. Papers analyse distributive justice, i.e. how to equitably allocate the burden globally of taking climate action, in light of differentiated responsibilities for harm caused and differing capabilities to adapt (Pan, 2003; Rao, 2014; Ji & Shu, 2015; Holz et al., 2018). Pan (2003) considers this dilemma also in an intranational context, noting the inequalities within countries, with Rao (2014) exploring the dilemmas entailed in negotiating burden-sharing globally, while ensuring that the poor rather than the elite within countries gain from deferred emission reduction obligations for emerging economies. This concern with intranational equity intersects with the politics of negotiating burden-sharing internationally.

Since the 2015 Paris Agreement, justice scholars ask whether these distributive dimensions are being ignored in a collective action mode as climate governance enters an alleged **post-equity phase;** wherein issues of harm, responsibility and capability are left to bottom-up, nationally determined approaches (Gupta, 2019; Klinsky & Gupta, 2019). This ensures that these contested issues get side-lined in favour of what is achievable within a neo-realist and neoliberal world. Increasingly countries describe whatever they do as ‘fair’ in their Nationally Determined Contributions (NDCs). Negotiating shared criteria for fairness have been shelved (Winkler et al., 2018), despite proposals on how the 1.5 °C target *can be* fairly shared (Holz et al.,, 2018) and how differentiated approaches can catalyse new and ambitious climate action (Chan et al., 2018). Another symbol of a post-equity world is the shift from the right to development to the watered-down right to promote sustainable development in the Climate Convention of 1992 (Gupta & Arts, 2018).

Increasingly, justice scholarship interrogates the equity implications of various approaches to meet the Paris Agreement’s targets. Flegal and Gupta (2018) question whether risky geoengineering options are being promoted as equitable. Dooley and Kartha (2018) show that expectations from negative emission technologies are too high and dependence on them risky. Gupta and Arts (2018) argue that the right to development is being misused to export fossil technologies to the Global South. Faran and Olsson (2018) show that cost–benefit analyses of bioenergy with carbon capture and storage technologies distract attention from how and which risks are being externalized. Together, these contributions question: (a) the presumption that realizing 1.5 ℃ in a just manner is achievable *without* radically changing economies and societies; and (b) whether equity considerations are being evoked to justify contested or speculative climate action such as geoengineering. The procedural, distributive and corrective justice consequences of these approaches remain shrouded in uncertainties.

Going forward, will justice or post-equity considerations be prioritized in scholarship and practice? Lahn (2018) tackles this by asking whether equity issues need to be politicized (‘heating up’) or depoliticized (‘cooling down’, i.e. through privileging technocratic processes and expert-driven scientific consensus), and which strategy works better and for whom. He provides two contrasting examples. The ostensibly cooled down Bali Box (Box 13.7, IPCC FAR, 2007) presented emission sharing between countries as a ‘neutral’ scientific story; developing countries cited this to demand action from rich countries, not realizing that this also required significant action from them. In contrast, the heated up Civil Society Equity Review divided countries into leaders and laggards and called on laggards to do more. Lahn supports ‘heating up’ equity discussions to promote accountability. However, will the architecture of the post-Paris climate regime, with its privileging of bottom-up NDCs and deference to sovereignty, allow equity considerations back into the political debate? We think this is both necessary and inevitable, if we are to achieve the 1.5 ℃ target.

### Water: Justice through principles, participation and counter-hegemony

INEA has over 50 articles on water, the no harm principle (see Sect. 2.2), securitization, and hegemony. We discuss here discursive elements, access and allocation and treaty design. As water is scarce in many contexts, there is conflict over water sharing and pollution making water a *political good* (Schouten & Schwartz, 2006). Some papers show how concepts such as integrated water resources management and cooperation obscure justice issues, the hegemonic nature of treaty negotiations (Gerlak & Mukhtarov, 2015; Zeitoun & Mirumachi, 2008) and/or sub-national social-ecological issues (Fox & Sneddon, 2007). Counter-hegemonic strategies are recommended (Zeitoun et al., 2011, 2017) to promote justice. Others refer to how states are securitizing and nationalizing water through making water an urgent issue, creating institutions to use and protect water infrastructures, excluding stakeholders, and short-cutting procedures to deal with drought and other crises (Fischhendler, 2015; Fox & Sneddon, 2007; Urquijo et al., 2015; Zikos et al., 2015).

On issues of access—human rights and the SDGs promote access to water and sanitation services. Some articles show how court cases are strengthening the right; the state of knowledge on access and allocation (Hurlbert, 2020); and the need to deliver water, energy and food as a ‘triplet’ (Sharma & Kumar, 2020). On allocation via treaty/policy design, articles focus on fairness through transparency and data exchange (Gerlak et al., 2011) and principles of water governance (e.g. equitable and reasonable utilization, stakeholder participation, polluter pays; Conti & Gupta, 2016). While these principles may promote justice, they are difficult to implement. For example, France refused to apply the polluter-pays principle on chloride pollution in the Rhine vis a vis the Netherlands (Dieperink, 2011). Public private partnerships to solve water access problems are contested as the return on investment for much of water use is low (Tecco, 2008).

In sum, the papers assess existing water governance instruments and question whether discursive, substantive or procedural power marginalizes justice issues. They promote the use of substantive and procedural principles and counter-hegemonic strategies to promote justice.

### SDGs and justice: strong on access, weak on allocation

INEA increasingly publishes on the SDGs. The 2030 Agenda requires that the 17 thematic Goals be implemented synergistically to ensure that social–ecological justice issues are not sacrificed for other goals (Boas et al., 2016). It has prioritized access with many ideal social goals, but it is weak on allocation. First, although it aims to reduce inequality, this is not elaborated in terms of resource allocation. Second, harm and liability are not mentioned. Third, it returns to ‘full permanent sovereignty’ which undermines the call for partnerships and cooperation.

Nevertheless, how was this modest systemic step towards synergy taken? This was possible because countries negotiated in troikas (in groups of three representing different kinds of countries) which broke the North–South divide; a deep stocktaking process built mutual trust; and the co-chairs led by monitoring the evolution of the text (Chasek & Wagner, 2016). Millions were engaged in the world’s largest involvement of bottom-up voices (e.g. ‘A Million Voices: The World we Want’; and ‘My World Survey’; Gellers, 2016). However, the process was not inclusive enough (Sénit et al., 2017) and the justice framing was narrow (Sénit, 2020) demonstrating compromises between different arguments and interests (Gupta & Vegelin, 2016).

## Going beyond: implications for the 2030 Agenda

### Introduction

Having discussed general trends in INEA justice-related scholarship, we now examine the implications for interpreting and implementing the 2030 Agenda and beyond. INEA articles have progressed from a cautious pragmatic approach of describing what is happening in international institutions and showing their distributive impacts, to calling for research focusing on access and allocation, to explicitly assessing justice issues. Justice issues have moved from being marginal to mainstream. This trend is mirrored in scholarship elsewhere. For example, ESG scholars rebutted Prof. Keohane’s statement that addressing climate change effectively required setting aside issues of equity in *Global Environmental Change* (Klinsky et al., 2017). The editorial board of *Third World Quarterly* resigned after the publication of Bruce Gilley’s 2017 article on the benefits of colonialism. ‘Planetary justice’ is being advanced within ESG scholarship (Biermann & Kalfagianni, 2020; Kashwan et al., 2020), to balance the debate around the need to remain within planetary boundaries. There is a changing mood in academic enquiry, and we make four arguments.

### Just approaches are needed for solving environmental problems

Is justice an issue of morality, legality or is it becoming a necessity for human survival? Is justice a choice? Through history, justice has been on the agenda, but has often been seen as a choice. Revolutions have often addressed injustices and put new values on the table—such as equality, liberty and fraternity. We argue here that justice is no longer a choice at the global level; without a just approach—the whole boat sinks. Hardin’s life boat ethics (1974) argued against: a just approach; recognizing human rights; aid; migration; and compensation for native Americans, arguing that then the proverbial boat would sink in environmental disasters. However, if there are more people outside the boat, they will pull down the boat if they cannot get in.

In the Anthropocene, we are destabilizing the natural systems that have made the Earth habitable for us. The Global Environment Outlook (UNEP, 2019) and Making Peace with Nature (UNEP, 2021) confirm that we have a decade to correct our behaviour to avoid setting into motion processes that we cannot reverse. If industrialized countries understand this urgency, they may be willing to alter their production and consumption patterns. However, in an unjust world, there is no guarantee that the rest will be willing to sacrifice the use of their fossil fuels, land, water and minerals. Using border tax adjustments in Europe will not force the rest—given that the rest is so large. Thus, even if the North cleans up its act, it cannot force the rest to do so—especially as they have not historically caused the problem and are at the receiving end of environmental impacts. Moreover, as the first impacts hit, the death and displacement caused will have repercussions on the North. The COVID-19 crisis provides a small taste of what is to come. Hence, *without a just approach to anchor SDG implementation, we cannot stay within planetary boundaries.*

### Collective action and social practice models need to merge in the search for just solutions

Institutions are formed by behaviour that conforms to collective action or social practice models (see Sect. 2.1). Young argued that we should study why one prevails over the other and unite these approaches. INEA papers reveal the dominance of the collective action models with many papers analysing how treaties maximize benefits for individual states and actors and prioritize market-based approaches for solving problems (see other papers, this Special Issue).

Such approaches promote thin market ‘justice’ which benefits big and rich actors; this may reproduce injustices. When policies are implemented within the social practice model, there is a greater chance of achieving ‘justice’ although there will be winners and losers. However, here the losers will be those who have made profits in the past and have the broadest shoulders to carry the burden. Table 1 links the institutional models to justice.

**Table 1:** Institutional Models and Justice

	Collective action models	Social practice models
Logic	Rational assessment of costs and benefits for actors: *This may underestimate the existential costs for the poor and externalize social and ecological costs*	Assessment of appropriate norms that define the identity of society (e.g. democratic, just): *What is just is defined by society; it evolves over time and can reflect court judgements and social movements*
Focused on	Agents: *This may prioritize the big actors (e.g. large farmers, MNCs) over small ones (small holders, SMEs)*	Structure: *This may enable a balance between the role of different actors in a society*
Context, driving factors	Deregulation and markets, capitalism: *This may lead to markets shifting scarce resources away from basic needs to producing luxury goods with higher returns on capital and resources. The environment may be externalized*	Capitalism, unregulated markets and GDP growth may have caused the problems: *Recognition may lead to regulating markets to ensure social and ecological aspects are not undermined by markets*
Instruments	Carrots and sticks to change behaviour: *This may require large carrots and sticks for rich and powerful actors; a system based purely on carrots and sticks is not sustainable or affordable*	Carrots and sticks need to be complemented by suasive (persuasive) measures that promote norm creation, diffusion and normative forcing; internalization of norms. *A judicial policy mix is necessary for a just transformation*
Type of justice achieved	Achieved via rational behaviour of (powerful) agents and markets: *Conservative justice, market justice, limited market justice; procedural justice has low benefits for the marginalized*	Achieved via socialization and institutionalization of norms of behaviour: *Conservative to ideal justice; corrective and distributive justice; procedural and substantive justice*
Relation to access and allocation	Collective action models initially ignored access issues. Following the 2030 Agenda*, they increasingly recognize access; but not yet issues of allocation*	Social practice models are recognizing that: *Poverty, vulnerability and marginalization are not intrinsic, but created; and access cannot be addressed without addressing allocation issues*

Increasingly papers show that market mechanisms and international treaties are failing to address environmental problems. The carrots and sticks approach, typical for collective action models which depend on incentivizing actors to change their behaviour, is insufficient. Who can raise the resources to incentivize actors worldwide in the Anthropocene to change their behaviour? Law scholars recognize that sticks are unsuccessful if the bulk of the population disagree in a democracy. This is why we need to merge the collective action and social practice models: possibly collective action should function within a broader social practice model. The social practice models should focus on creating the appropriate justice norms and ‘normative forcing’, the suasive (persuasive) instruments to educate people to convince them of the need for urgent change (e.g. labelling and information campaigns that eating meat is bad for human health and the environment), and instruments for addressing inequality—such as tax justice (to address the appropriation of wealth) and corrective justice (to ensure that those who make profits by externalizing risks are held accountable for their acts). Within a broad framework of social practice models and regulation, markets may function and carrots and sticks devised. *Thus collective action models need to be embedded within a large social practice model*.

### Access issues are becoming politically palatable, but allocation issues are much more contested

With the 2030 agenda, access issues are becoming politically palatable but allocation issues remain more contested. Social goals (e.g. end poverty and hunger) have been prioritized at the same level as environmental and economic goals. This has prioritized issues of access. INEA has barely covered social issues, but the 2030 Agenda has justified examining issues of access in relation to the environment as the goals are indivisible. Thus market mechanisms to address environmental issues—such as the clean development mechanism, reducing emissions from deforestation and degradation, and border tax adjustments—need to be tested against their impacts on access as in the Special Issue on Access and Allocation.

However, when we examine resource allocation—discussed within INEA in relation to water (where hydro hegemony played a role) and climate change (where we see a shift to a post-equity bottom-up approach), and allocation of risks (focusing on loss and damage, liability, and the no harm principle), these remain highly contested. Much of allocation research has been undertaken within the collective action model, taking the rules as given and then assessing the outcomes. Contestation of allocation issues were not resolved in the 2030 Agenda – despite the inequality Goal 10. Thus the SDGs and recent scholarship emphasize just access over just allocation.

### However, just access is not possible without just allocation

For policymakers from industrialized countries and for scholars in the collective action tradition post 2015, it makes sense to focus on access and not on allocation or on allocation via the market or market mechanisms. However, *although we do not wish to undermine efforts to achieve minimum access*, we submit that INEA papers show that fair access is not possible without fair allocation.

First, the scholarship shows that vulnerability is neither innate nor intrinsic but politically created, constructed and performed, often through allocation rules. Socio-economic vulnerability may arise from existing institutions such as market distribution, and ecological vulnerability may arise from ecological damage (e.g. climate change, biodiversity loss, water pollution, land degradation, zoonosis) which create new existential challenges (Grecksch & Klöck, 2020; Ivanova et al., 2020).

Second, if markets allocate scarce resources, the price goes up, aggravating inequality. Trade and investment regimes, while aiming to improve the lives and livelihoods of people, often achieve these goals at the cost of vulnerable people, sectors and countries (Gonenc et al., 2020; Scobie, 2020). Resource extraction in countries with poor labour/environmental regulations leads to socio-ecological exploitation of the poor (Scobie, 2020), despite rules on access and benefit sharing in the biodiversity regime. Resource use in a market allocation model ensures that those who can afford resources will buy them up in a market where resource scarcity pushes up prices, thus pushing out those who cannot afford to pay for these resources or who have insecure access through unrecognized tenure rights (e.g. leading to land (and water) grabbing; Azizi, 2020). If market mechanisms are combined with certification schemes, than this excludes the poor who cannot afford to participate in such schemes (Gonenc et al., 2020). Markets in combination with perverse subsidies affects access. Subsidies in the Global North affect poverty in the Global South—as they encourage overfishing and overuse of fossil energy (Scobie, 2020). The policy climate allows western governments to sell old plastic and electronic goods to the South while also transferring the burden of waste management to them (Cotta, 2020). Global asset managers invest in scarce resources exacerbating dispossession, land and water grabbing, while some technologies both externalize risks (see Sect. 4.1) and substitute for existing social capital— labour—concentrating wealth while negatively affecting fair access and allocation (Sharma & Kumar, 2020). There is growing evidence of divested fossil fuel shares being sold to developing countries, transferring the potential losses of stranded assets to them.

Third, even within the world of international aid (philanthropy and government aid), there is a tendency to mask the underlying causes of global inequality while focusing symbolically on access issues and often only temporarily (Scobie, 2020). For example, public money is used for export credit to support western producers to invest in fossil fuel in the South which leads to a fossil fuel lock-in and debt in the latter (Gupta et al., 2020).

Fourth, some scholars argue that procedural justice (access to information, participation, representation and courts) will enable substantive justice. However, procedural justice may lead to unjust outcomes as the powerful influence policy processes (Azizi, 2020; Gonenc et al., 2020) and empowering the powerless may require more than just recognizing equality of opportunity (Kelly, 2010; Ross, 2015). Much of justice literature focuses on how, despite good intentions underpinning access and allocation mechanisms, they do not deliver equitable and just outcomes (Coolsaet et al., 2020). However, there are also empirical stories of success in securing both, where biodiversity aid and crowd funding have enhanced species protection (Scobie, 2020).

Our screening of the INEA justice scholarship shows that while the separation between access and allocation is justified from what we observe in the global arena and as an analytical device, access goals cannot be met without drastically revisiting allocation issues.

## Conclusions

This article has traced the arc of INEA justice scholarship, showing how it has moved from empirically assessing how institutions are negotiated, to directing attention to justice issues as access and allocation, to understanding how scholarship and practice on access and allocation fit within different empirical contexts and epistemologies of justice.

The need to secure access was recognized in human rights treaties but half-heartedly implemented. The 2030 Agenda has made this a legitimate part of international policy and the Goals are formulated in aspirational terms, even if the targets are more ‘conservative’. With regard to allocation, although some articles talk of fair allocation of resources, most papers emphasize the use of market mechanisms to achieve allocation (‘conservative justice’). Although there has been talk of allocating responsibilities based on common but differentiated responsibilities, policy processes are moving into an alleged post-equity phase, where equity questions are sidelined or removed from international debate. While the allocation of risks/harm has a history at national and transboundary (e.g. in water agreements) levels, at the global level, this principle is being marginalized possibly because the activities that cause harm are ‘legal’ and because those causing the harm are powerful. While not causing harm to other countries was subsumed under the sovereignty principle at Stockholm in 1972 and Rio in 1992, the 2030 Agenda has reincarnated the ‘full permanent sovereignty’ principle. Corrective justice has become a casualty of this process. Perhaps court cases, counter-hegemony, stakeholder participation and social movements can reverse this trend.

We conclude that: (a) justice is more than an issue of morality and legality, it is becoming necessary for living within planetary boundaries in the Anthropocene; conservative, reformative justice will not be enough; and procedural justice needs to go beyond equal treatment. (b) Collective action approaches will need to be embedded within social practice models to effectively address global problems. (c) While issues of access are becoming politically acceptable, issues of allocation of resources, risks and responsibilities remain contested. However, (d) without fair allocation, we will not be able to guarantee access. If allocation is undertaken through markets within a neo-liberal capitalist process, this exacerbates the marginalization of the poor. If harm is not redressed, those causing harm will continue doing so. Achieving access will become ‘*dweilen met de kraan open*’ (mopping the floor while the tap remains open) as the Dutch say; the nearest equivalent in the English language is swimming against the tide. It is important to strategically separate access and allocation—because only then does it become clear that both are needed. Finally, (e) given that the driving forces of environmental degradation and inequality arise primarily from the economic, political, legal and cultural systems, justice requires transforming these systems in order for planetary justice to be achieved. These are also our messages for the Stockholm plus 50 Conference in 2022.

## Abbreviations

ESG: Earth System Governance
IDGEC: Institutional Dimensions of Global Environmental Change
IHDP: International Human Dimensions Programme
INEA: International Environmental Agreements: Politics, Law and Economics
SDGs: Sustainable Development Goals
UN: United Nations
UNGA: United Nations General Assembly
